# Evaluation of the practice of surgical antibiotic prophylaxis in a Zonal Referral Hospital in Mbujimayi, Democratic Republic of the Congo (DRC)

**DOI:** 10.1186/s12893-023-01926-7

**Published:** 2023-02-04

**Authors:** Hervé Tshikomba Mbuyamba, Crispin Mukendi Muamba, Séraphin Katulondi Binene, Séverin Akinja Uwonda

**Affiliations:** 1 Department of Surgery, Université Officielle de Mbujimayi (UOM), Mbujimayi, Democratic Republic of the Congo; 2 Department of Surgery, Muhimbili University of Health and Allied Sciences (MUHAS), Dar es Salaam, United Republic of Tanzania; 3 Department of Surgery, Bonzola Zonal Referral Hospital, Mbujimayi, Democratic Republic of the Congo

**Keywords:** Surgery, Antibiotics, Prophylaxis, Compliance, Evaluation, Bonzola Zonal Referral Hospital

## Abstract

**Background:**

Surgical Antibiotic Prophylaxis (SAP) is helpful in preventing patients from developing Surgical Site Infections (SSI). In Mbujimayi, the documentation on the practice of SAP is outdated and inadequate. The last study was conducted more than 5 years ago. This study aims at assessing the compliance of the practice of antibiotic prophylaxis in the surgical and obstetrics-gynecology departments of the Bonzola Zonal Referral Hospital (BZRH) compared to the international standards.

**Methods:**

A prospective observational study was conducted from March 2020 to March 2021 involving 324 surgical patients who received antibiotic prophylaxis. Interventions were assessed as “compliant” if all the variables individually complied with the criteria for antibiotic prophylaxis use.

**Results:**

Three hundred and twenty-four patients were enrolled in this study. Compliance was found to be 87.35% for the indication for administration; 0.31% for the choice of the molecule; 3.65% for the time of the first administration; none for the duration of antibiotic prophylaxis. Therefore, the overall compliance was nil. This study shows a significant gap when the current practice in Mbujimayi town is compared to the recommendations of international societies.

**Conclusion:**

SAP is often indicated in accordance with international recommendations in Mbujimayi. However, the choice of the molecule, the dosage, the time of first administration and the duration of SAP deviate from them. Thus, the compliance of SAP is nil.

## Background

Surgical Antibiotic Prophylaxis (SAP) is defined as the administration of antibiotics before contamination by surgical incision has occurred and is given with the intention of preventing infection [1, 2]. The SAP helps to reduce the risk of postoperative infections [2, 3]. It should be used appropriately in surgical settings.

A study conducted in the United States of America (USA) from 2012 to 2018, showed that infectious postoperative complications trends are decreasing. However, postoperative infections are still ranked first out of all postoperative complications with various frequencies: sepsis (1.6%), superficial surgical site infection (1.5%), organ/space (1.3%), urinary tract infection (1.3%) and deep surgical infection (0.5%) [4].

In Africa, the Surgical Site Infections (SSI) are the most common care-associated infections [3]. A meta-analysis study reported an incidence of 18.6% for SSIs in Sub-Saharan Africa [5]. Ivory Coast reports a frequency of 8.6% as against 13.4% in Bamako (Mali) [6]. Another study reported an incidence risk of 10% in Ghana [7]. A high frequency was reported in Tanzania where SSIs occurred in 35.6% operated patients at Muhimbili National Hospital [8]. The incidence of postoperative infections is still alarming in Africa [7].

In the Democratic Republic of the Congo, a study was conducted on SAP at the Referral Hospital of N’djili by N’sinabau, in 2020. He reported an overall compliance of 33% [9]. During the same year, another study was conducted in Butembo by Bunduki et al.; they reported a SAP compliance rate of 18.1% [10].

Mukenga et al. conducted a similar study from January 1 to June 30, 2014, at Dipumba Hospital in Mbujimayi, in the Province of Kasai Oriental, DRC. They found that the use of SAP did not comply with international recommendations with a compliance rate of about 4.8% [11].

Despite advances in asepsis management, SSIs remain a public health problem even in developed countries. For example, the incidence rate of SSIs varies between 3.6% and 4.2% in Belgium and 2.8% in France [12].

Any surgical procedure carries a risk of SSI which must be as low as possible. Nevertheless, the risk can never be zero since the skin barrier has been crossed. Germs also find a favorable environment for their proliferation due to favorable factors such as lasting surgical procedure, stage of wound contamination, anemia, ischemia, hematoma, implants, long stay at hospital… [5]. SSIs are feared by surgeons because they jeopardize the surgical procedure outcome. Thus the use of SAP leads to the reduction of SSIs [2]. However, the use of SAP should comply with evidence-based recommendations.

Research works on SAP in Mbujimayi are very rare. The only known study on this subject dates back to 2014[11]. Therefore, this study aimed to assess the current use of SAP in Mbujimayi in comparison to the international standards.

## Methods

### Design and setting of study

It was prospective and observational study carried out from March 2020 to March 2021 in the surgical and obstetrics and gynecology departments at the BZRH, located in Mbujimayi, province of Kasai Oriental, in DRC. It is a tertiary-level hospital that has approximately the capacity of 150 beds for the surgical department and 100 beds for the obstetrics and gynecology department. It is under the authority of the Bakwanga Mining Company (MIBA).

### Target population

The target population was constituted of all patients operated on in the surgical and obstetrics and gynecology departments during the study period.

### Selection criteria

All patients whose surgical procedure was classified as Altémeier I or II and having undergone antibiotic prophylaxis were included in this study according to *Société Française d’Anesthésie et Réanimation* (SFAR) recommendations [13].

All the operated patients who were operated elsewhere but referred to the BZRH for postoperative follow-up and those whose surgical wound corresponded to classes III and IV of Altémeier were not included in the study.

### Sample size and data collection

**Fig. 1:**
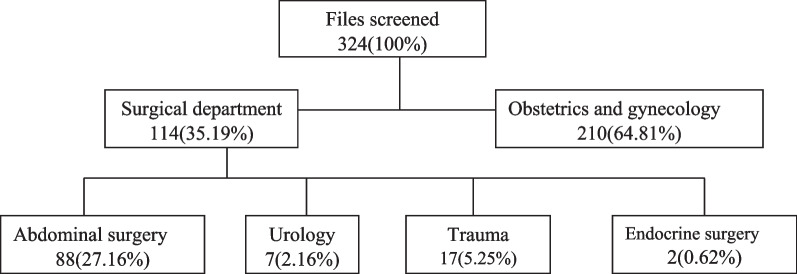
Breakdown of patients by departments

The sample size was estimated using Fischer’s formula in a simple population, assuming that the compliance of antibiotic prophylaxis is 33%, within a 95% confidence interval and 5% marginal error. Thus, the minimum size was 324 (Fig. 1).

The data were collected using a pre-established form. The important data were taken from the medical files of the patients (consultation form, anesthetic form, and procedure notes). The general and main data relating to antibiotic prophylaxis collected were: indication for the use of SAP, choice of antibiotic, time of administration of the first dose and route of administration, initial dose, and the duration of SAP. The general variables were: sex, age, Altémeier class, nature of the surgical intervention (emergency or elective surgery), duration of the surgical operation, ASA score and duration of hospitalization.

SAP compliance was assessed considering criteria developed by the National Institute for Health and Care Excellence (NICE) [14], Stanford Health Care (SHC) [15], and SFAR [13] adapted to our setting. These guidelines recommend that SAP should be given to patients before Altemeier I surgery and Altemeir II surgery. The antibiotic should be given within 30–60 min prior to the incision. It is recommended to give a repeat dose of antibiotic prophylaxis when the operation is longer than the half-life of the antibiotic given. Antibiotic treatment is given to patients having surgery on a pre-existing dirty or infected wound [13–15].

### Operational definitions

The six variables relating to the use of SAP were evaluated. Interventions were assessed as “compliant” if all the variables individually complied with the criteria for antibiotic prophylaxis use. If one or more variables did not comply with the prescription criteria, the intervention was then considered “non-compliant”. The variables are: indication of SAP, initial dosage and administration route, time for administration, duration of utilization and starting time of SAP [16, 17].

Surgical wounds have been classified according to Altémeier’s classification into four classes as recommended by CDC in clean, clean-contaminated, dirty and dirty-contaminated [18].

The patients were classified according to ASA score as follows: normal healthy patient (ASA I), patient with mild systemic disease (ASA II), patient with severe systemic disease (ASA III), patient with severe systemic disease that is a constant threat to life (ASA IV), moribund patient who is not expected to survive without the operation (ASA V) and a declared brain-dead patient whose organs are being removed for donor purposes (ASA VI) [19].

### Data analysis

The data was encoded in Excel 2010 software and then analyzed using Epi Info version 7 software. We calculated the frequency, percentage and mean. The results are presented in the form of tables and figures.

### Ethical considerations

The protocol of this study was approved by the ethical board of the Université Officielle de Mbujimayi. Confidentiality and anonymity of patients and patient information were maintained. The informed consent was obtained from the patient before the collection of any information.

## Results

### General characteristics

#### Demographic characteristics

The female sex was much more represented in this study with 269 out of 324 patients or 83.03%.

Patients aged between 20 and 30 years old were the most represented with 129 out of 324 patients or 39.81%. The mean age of the patients was 40 years with extremes of 17 and 84 years excluded (Table 1).

**Table 1:** Distribution of patients according to their age

Age (years)	Frequency (n = 324)	%
< 20	46	14.20
20–30	129	39.81
31–40	74	22.84
41–50	26	8.03
> 50	49	15.12

#### Characteristics of surgical interventions

Most of surgical procedures were classified Altemeier II in 273 patients out of 324, or 84.26%. One hundred and sixty-nine operations were carried out as emergency procedures out of the 324 surgical procedures, or 52.16%. The duration of the intervention was less than 60 min in 64.81% of patients (Table 2).

**Table 2:** Distribution of patients according to the characteristics of the surgical procedures

Characteristics of procedures	Frequency (n = 324)	%
Altemeier’s classification		
I	51	15.74
II	273	84.26
Character of surgical procedure		
Emergency	169	52.16
Elective	155	47.84
Duration of the intervention (minutes)		
< 60	210	64.81
60–120	102	31.49
> 120	12	3.70

77.16% of the patients were classified as ASA I. The majority of operated patients, 70.68%, spent between 7 and 14 days at hospital after surgery (Table 3).

**Table 3:** Distribution of patients according to ASA score and length of hospitalization

Variables	Frequency	%
ASA Score		
ASA I	250	77.16
ASA II	54	16.67
ASA III	18	5.55
ASA IV	2	0.62
Duration (days)		
< 7	10	3.09
7–14	229	70.68
15–25	52	16.05
26–35	17	5.25
> 35	16	4.93

N = 324

### Antibiotic prophylaxis data

The indication for antibiotic prophylaxis was compliant in 87.35% of prescriptions. The antibiotic was administered to the patient after surgical procedure in 96.50%. The recommended initial dose was administered to most of the patients once (99.07%). SAP had lasted more than 48 h in 100% of operated patients and were administered intravenously in all patients (Table 4).

**Table 4:** Distribution of operated patients according to the practice of antibiotic prophylaxis

Practice of antibiotic prophylaxis	Frequency (n = 324)	%
Indication for SAP		
Compliant	283	87.35
Non compliant	41	12.65
Time of administration		
After the procedure	312	96.50
At the beginning of the procedure	12	3.50
Initial dose		
Once	321	99.07
Repeat dose	3	0.93
Duration (hours)		
> 48	324	100.0

Ceftriaxone was the most prescribed antibiotic, often in combination with gentamycin, metronidazole, and ampicillin (Table 5).

**Table 5:** Distribution of operated patients according to antibiotics received for prophylaxis

Antibiotics	Frequency (n = 324)	%
Ceftriaxone	113	34.88
Metronidazole + Ceftriaxone	110	33.95
Metronodazole + Ampicillin + gentamycin,	74	22.84
Metronidazole + Ceftriaxone + gentamycin,	7	2.16
Metronidazole + Ampicillin	6	1.85
Ampicillin + gentamycin,	5	1.54
Metronidazole + gentamycin, + Ampicillin	5	1.54
Ciprofloxacin + Metronidazole	2	0.62
Co-amoxyclav	1	0.31
Ampicillin	1	0.31

### Overall assessment of antibiotic prophylaxis

The overall assessment of the practice of SAP was nil because all the assessment criteria were not respected in any operated patient (Table 6).

**Table 6:** Distribution of operated patients according to the overall assessment of the practice of antibiotic prophylaxis

Variables	Assessment of SAP	
	Compliant n (%)	Non compliant n(%)
Indication	283 (87.35)	41 (12.65)
Time of administration	12 (3.50)	312 (96.50)
Initial dose	3 (0.93)	321 (99.07)
Duration of antibiotic prophylaxis	0 (0.00)	324 (100)
Choice of molecule	1 (0.31)	323 (99.69)

Total = 324

## Discussion

### General characteristics

#### Demographic characteristics

Women were more represented in the current study with about 83.03%. These results are similar to those found by Mukenga et al. [11] in Mbujimayi, in 2017, who had reported 66.1% of operated cases being women. The same trend was reported by N’sinabau et al. [9] in Kinshasa, in 2019; they reported 83.1% of women in their series. This could be explained by the fact that many patients were admitted into the obstetrics and gynecology department in all the series.

The most common age of the patients in the series was between 20 and 30 years old (39.81%). The mean age in our series was 40 years old with extremes of 17 and 84 years excluded. These results are close to those found by Mukenga according to which operated patients had the mean age of 35 years with extremes of 18 and 80 years old in his study [11].

#### Characteristics of surgical interventions

Most of surgical wounds were classified as Altermeier II in 84.26%, while in Dembele’s series [20], this wound class represented 58.7%. Our rate is far higher than that mentioned in Dembele's series. This could be explained by the fact that we have many patients from obstetrics and gynecology department who are mostly of the Altemeier II class. However, it should be noted that, most of the surgeries were performed as emergency procedures in our series, 52.16% approximately. Emergency procedures were found to be one of factors favoring the occurrence of SSI in different settings [21–23].

Most patients were operated on for less than 60 min (64.81% of cases). This could be explained by the emergency nature of most of the surgical interventions, especially in the department of Obstetrics and Gynecology. The impact of the duration of surgery on the occurrence of surgical site infection has been mentioned in some studies [5, 24, 25]. The risk would be particularly increased for surgical operations lasting more than two hours [26].

Operated patients were classified as ASA I in 77.16% in our series, whereas Dechoux reported 66.1% of patients classified as ASA I in her series [26]. The postoperative hospital stay varied between 7 and 14 days in 70.68% of cases in our study. This is close to Dechoux’s result regarding this aspect**.**

### Antibiotic prophylaxis data

The indication for SAP was considered to comply with the international recommendations in 87.35% of cases in our series. This rate is significantly higher than those reported by Mukenga (53.2%) [11] as for Arquès [27] and Majjad [28], they reported the result similar to ours [27]. However, the SFAR recommendations do not cover all clinical situations. Many acts have not been subject to scientific evaluation. In the absence of recommendations for a specific subject, practitioners may or may not choose to prescribe prophylactic antibiotics by getting as close as possible to similar conditions [13].

The administration of the first dose was mostly done after the surgical procedures (96.35%) in our series. This result is similar to that of Mukenga [11] who reported 82.3% administration of the 1st dose after surgery. This would be explained by the emergency of the surgical operations which predominates, but also the socio-economic level of the surgical population of our environment.

In the current study, 99.7% of patients had received a non-compliant dosage. The same observation was made by Mukenga who reported 85.5% [11]. According to studies conducted elsewhere on the same subject, the dose was compliant in 89% of operations in the series of Van Kasteren [29] and 100% in that of Vaisbrud [30]. The lack of knowledge on the practice of antibiotic prophylaxis in our environment could be the reason for this high percentage of non-compliance with the dose criteria.

The duration of SAP exceeded 48 h in 100% of cases, whereas Mukenga had reported 62.9% in his series [11]. Vaisbrud had found that the duration of SAP was less than 24 h in 91% of his series [30] while Arquès had reported a shorter duration in 78.5% of antibiotic prophylaxis [27]. The precarious aseptic conditions in our environment could explain the continuation of the antibiotic beyond the recommended time [11].

The choice of antibiotic complied with the standard in only 0.31% of cases in our study. This choice was outside the scope of recommended molecules, especially in terms of broadening the spectrum. However, it is recommended that the antibiotic prescribed must include, in its spectrum of action, the most common bacteria responsible for SSI [10, 31, 32]. In the series studied, gentamycin, ampicillin and ceftriaxone were used more than other molecules, especially in combination with other antibiotics. However, Arquès [27] has reported a compliance rate of the choice of antibiotic clearly higher than ours (89.8%). A study conducted in Australia, based on the Australian consensus, reported a compliance rate of 53.3% [33]. According to SFAR recommendations, aminopenicillins can be used, but in combination with a beta-lactamase inhibitor [13]. The prescription of 3rd generation cephalosporins is not suitable for antibiotic prophylaxis because these drugs are expensive and their use leads to the emergence of mutants resistant to these useful drugs for curative treatment [10]. Lack of knowledge of the recommendations on the choice of antibiotics to be used as first-line treatment in our setting would justify this non-compliance.

This study presentes some limitations. It was conducted exclusively at the BZRH because of the hospital’s importance in the surgical management of patients in the city of Mbujimayi. However, extending this study to other hospitals could provide increasingly reliable data on the practice of SAP in Mbujimayi. It is also necessary to conduct a further study to elucidate the determinants of non-compliance of SAP with evidence-based guidelines in the city of Mbujimayi.

## Conclusion

SAP is often indicated in accordance with international recommendations in Mbujimayi. However, the choice of the molecule, the dosage, the time of first administration and the duration of antibiotic prophylaxis do not comply with them. Thus, the compliance of SAP is nil. It is therefore important to address relevant measures in order to reverse the trend.

Anyway we recommend BZRH and Authorities to elaborate local guidelines adapted to the surgical environment and to invest in continuous training of medical staff on how to use of antibiotics.

## Data Availability

All data generated or analyzed during the study are included in this published article.

## Abbreviations

ASA: American Society of Anesthesiologists
BEBUC: Bourse d’Excellence Bringmann aux Universités Congolaises
BZRH: Bonzola Zonal Referral Hospital
CDC: Center of Disease Control
DRC: Democratic Republic of the Congo
MIBA: Minière de Bakwanga
NICE: National Institute for Health and Care Excellence
SAP: Surgical Antibiotic Prophylaxis
SFAR: Société Française d’Anesthésie et Réanimation
SHC: Stanford Health Care
SSI: Surgical Site Infections
USA: United States of America
UOM: Université Officielle de Mbujimayi
